# Capacity Bounds for Dense Massive MIMO in a Line-of-Sight Propagation Environment

**DOI:** 10.3390/s20020520

**Published:** 2020-01-17

**Authors:** Felipe A. P. de Figueiredo, Claudio F. Dias, Eduardo R. de Lima, Gustavo Fraidenraich

**Affiliations:** 1 Instituto Nacional de Telecomunicações—INATEL, Santa Rita do Sapucaí 37540-000, MG, Brazil; 2 IDLab, Department of Information Technology at Ghent University—IMEC, 9052 Ghent, Belgium; 3 DECOM/FEEC–State University of Campinas (UNICAMP), Campinas 13083-852, Brazil; aplnx@decom.fee.unicamp.br (C.F.D.); gf@decom.fee.unicamp.br (G.F.); 4 Eldorado Research Institute, Campinas 13083-898, Brazil; eduardo.lima@eldorado.org.br

**Keywords:** massive MIMO, channel capacity, dense networks, outage probability

## Abstract

The use of large-scale antenna arrays grants considerable benefits in energy and spectral efficiency to wireless systems due to spatial resolution and array gain techniques. By assuming a dominant line-of-sight environment in a massive multiple-input multiple-output scenario, we derive analytical expressions for the sum-capacity. Then, we show that convenient simplifications on the sum-capacity expressions are possible when working at low and high signal-to-noise ratio regimes. Furthermore, in the case of low and high signal-to-noise ratio regimes, it is demonstrated that the Gamma probability density function can approximate the probability density function of the instantaneous channel sum-capacity as the number of served devices and base station antennas grows, respectively. A second important demonstration presented in this work is that a Gamma probability density function can also be used to approximate the probability density function of the summation of the channel’s singular values as the number of devices increases. Finally, it is important to highlight that the presented framework is useful for a massive number of Internet of Things devices as we show that the transmit power of each device can be made inversely proportional to the number of base station antennas.

## 1. Introduction

During the past years, we have been witnessing massive multiple-input multiple-output (MIMO) becoming an efficient and indispensable sub-6 GHz physical-layer technology for wireless and mobile networks. The embodiment of such technology was vital for the current 5G new radio (NR) interface [1]. The central concept behind massive MIMO is the use of a large antenna array deployed at base stations (BS) to simultaneously serve a large number of devices over the same time–frequency resources. In this way, the technique allows exploiting differences among the propagation signatures of the devices in order to perform spatial multiplexing [1]. Even though the massive MIMO technology looks quite mature, being adopted by new standards, it does not mean an end for research in this subject but just the beginning of unforeseen possibilities [2].

Current requirements for next-generation networks such as high bit rates, very low latency, high energy efficiency, and link robustness are not wholly met even by the current 5G solutions [3]. Thus, there are still several open challenges that need to be addressed by researchers.

One of the approaches used to increase throughput is by increasing the network density, that is, decreasing the size and increasing the number of cells in the same coverage area. As a result, the size of the cells is becoming smaller and smaller, and consequently, it is quite probable that wireless channels will be predominantly line-of-sight (LoS) [4,5]. Furthermore, the LoS characteristic becomes even more apparent as technology moves to millimeter-wave (mmWave) bands in order to fulfill the requirements for wider bandwidths (i.e., micro and femtocells) [6,7]. The mmWave bands have the potential to significantly raise user throughput, enhance spectral and energy efficiencies, and increase the capacity of mobile and wireless networks using the joint capabilities of the huge available bandwidth paired with the high multiplexing gains achievable with massive antenna arrays [8,9]. Above all, as the wavelengths of mmWave bands are extremely short, it becomes possible to add a huge number of antenna elements to a small area, which consequently helps in achieving the high multiplexing gains offered by the massive MIMO technology at both the BS and devices [10]. Additionally, the huge deployment of small cells, i.e., densification, renders the short-range mmWave technologies very useful. Therefore, the mmWave bands can be considered as one of the potential technologies to meet the requirements of 5G networks [11].

Densification is a natural process for wireless communication networks as the demand for connectivity increases. Thanks to smartphones, tablets, and the Internet of Things, wireless subscribers are using more network resources with no sign of a decrease in the demand rate. Operators need to add more capacity to their networks to continue handling all the traffic while providing the network speeds those users expect. An arbitrary coverage region can be expected to follow three different degrees of BS densification [2,12]: low-density, dense, and ultra-dense.

Starting from low density, the smart farming and rural broadband services provision (i.e., distant areas with low population density) poses a significant gap in the research body of massive MIMO nowadays [13,14]. In the sense of wireless services, it is essential to observe that most economically viable areas for agriculture are the plain terrains with few obstacles [15]. Thus, since the multi-path channel statistics of rural and distant areas are different from those found in urban centers, the wireless channel will likely be predominantly LoS in such areas [16,17].

For regions where high value goods are constantly under surveillance, a dense network is required to communicate with unmanned aerial vehicles (UAVs), also known as drones [18]. As with the other examples, these communications will probably be LoS-based as well. Additionally, in wireless and cellular communications, the probability that the devices have LoS to the BS is highly probable in many scenarios [19].

Among the approaches and opportunities cited here, the Internet of Things (IoT) for drones, sensors, automated processes, etc. [20] pose several unsolved research challenges. For example, in the near future, swarms of drones, sensors, and actuators will be omnipresent adding up to billions of devices [21,22,23]. In this ultra-dense scenario, these devices will be always moving around the cell at different speeds and positions with a dominant LoS link to the BS. The sum-capacity achieved by a BS serving a massive number of drones, which have a dominant LoS link to the BS and are constantly moving around the cell, is still an open issue.

As shown in [1], the huge gains in performance offered by the massive MIMO technology are only obtained when the spatial signature of the devices are mutually orthogonal. Differently from independent and identically distributed (i.i.d.) flat Rayleigh fading channels, in LoS scenarios, the spatial signatures are only determined by the position of the devices within the cell. This way, the power of the device-interference will continuously change as the devices randomly move around the cell. Therefore, there will be several independent realizations of the device-interference power within a short interval. Consequently, the devices experience several different realizations of the device-interference power during the transmission of a single codeword. For that reason, in this work we are interested in analyzing the achieved ergodic sum-capacity by averaging the instantaneous sum-capacity over all possible locations of the devices.

Therefore, in this work, we assume a dominant (i.e., pure with no multi-path) LoS environment in a massive MIMO scenario with favorable propagation serving a massive number of devices constantly moving within the cell. With this aspect in mind, the objective of this investigation is to find capacity limits concerning the number of devices, number of base station antennas, and signal-to-noise ratio (SNR). More specifically, given the aforementioned assumptions, the contributions of this work are as follows.

- Derive an analytical expression for the ergodic channel sum-capacity.
- Show that the transmit power of each device can be made inversely proportional to the number of BS antennas.
- Find analytical expressions for the upper- and lower-bound ergodic channel sum-capacities.
- Present expressions for the ergodic channel sum-capacity in low- and high-SNR regimes.
- Demonstrate that a Gamma probability density function (PDF) can also approximate the PDF of the summation of the channel’s singular values (also known as total power gain of the channel matrix) as the number of devices increases.
- Demonstrate that the Gamma PDF can approximate the PDF of the instantaneous channel sum-capacity in low- and high-SNR regimes.
- Demonstrate that the approximated PDF of the instantaneous channel sum-capacity can be used to calculate the outage probability in low- and high-SNR regimes.
- We extend the distance from favorable propagation measure defined in [1] for the ergodic sum-capacity case assumed in this work.

The remaining of this paper is organized as follows: Section 2 discusses some related works. Section 3 presents the system model adopted in this work, while Section 4 discusses aspects of favorable propagation in the current investigated conditions. Section 5 reviews the concept of favorable propagation. Section 6 presents the results with parameters from reference systems. Finally, we close our discussion in Section 7 summarizing our conclusions.

## 2. Related Work

In [19] the authors analyze the performance of the outage probability for the uplink of massive MIMO systems considering a uniform rectangular array (URA) and a pure random LoS scenario. They show that the outage probability increases logarithmically with the number of BS antennas due to the interference hardening effect.

The authors of [24] study the user-interference in a single-cell multi-user pure LoS massive MIMO system. They demonstrate that the distribution of the interference term in the expression of the uplink signal-to-interference-plus-noise ratio (SINR) can be approximated as a Beta-mixture when pure LoS channels and element spacing of half-wavelength are considered.

In [25], the authors assume a single-cell multi-user massive MIMO system with flat Rayleigh channels. They assess the distribution of the user-interference, which is modeled as a random variable, and the probability of outage for the downlink of the massive MIMO system adopting maximum ratio combining (MRC) precoding. They derive a distribution for the user-interference power and based on that they obtain an analytical outage probability equation.

In [26], the authors demonstrate that in single-cell multi-user massive MIMO systems the free-space nonfading LoS channels becomes asymptotically orthogonal as the number of antennas increases, i.e., the channel offers asymptotic favorable propagation, which is the most desirable scenario in terms of maximizing the sum-capacity of such systems. They also demonstrate that when a finite number of antennas is considered, the channels are not mutually orthogonal. Additionally, they also introduce the distance from favorable propagation measure, which is used to measure how favorable the propagation offered by the channel is, i.e., it measures the gap between the sum-capacity and the maximum capacity that is achieved when the channel offers favorable propagation.

In [27], the authors evaluate the impact of antenna element spacing of a uniform linear array (ULA) on the capacity of fixed point-to-point massive MIMO systems considering pure LoS channels. They demonstrate that the optimal spacing between antenna elements of ULAs, achieving full spatial multiplexing, can be reduced compared to the well-known element spacing criterion adopted in previous investigations.

The authors of [28], show that switching from i.i.d. flat Rayleigh fading to uniformly random pure LoS propagation results in no significant loss in user throughput performance. They demonstrate that for massive MIMO systems to perform well in LoS conditions, it is sufficient to selectively drop a small number of users from service. They additionally provide an algorithm for deciding which users should be dropped.

In [29], the authors study the potential of employing the Massive MIMO technology for communications with drones. They discuss the performance of the achievable uplink capacity in the case of pure LoS conditions. They determine the optimal antenna element spacing for ULAs and URAs that maximizes the ergodic rate achieved by a drone.

All the aforementioned works did not consider the mobility of the devices and the influence of favorable propagation in their analysis. The focus of our work is on analyzing the impact of devices’ mobility and favorable propagation on the ergodic sum-capacity and outage probability. Therefore, to the best of the authors’ knowledge, there is no work that studied the ergodic sum-capacity and outage probability performance of massive MIMO systems under pure LoS and favourable propagation for a scenario with devices randomly moving around the cell.

## 3. System Model

Here we present the channel model adopted in [1]. We assume a channel model with only free-space non-fading (i.e., pure) LoS propagation between the BS and the devices and that the BS is equipped with a ULA with the *M* antenna elements spaced of λ/2, where λ is the signal’s wavelength. We do not consider the existence of the mutual coupling effect among antenna elements. There are *K* single-antenna devices deployed randomly within the cell that simultaneously transmit data towards the BS through the same time–frequency resources.

Additionally, we also consider that all the *K* devices being served by the BS are located in the far-field of the antenna array at the angle $\theta_{k}$ as measured relative to the array bore-sight [1]. Therefore,

(1) $$\begin{array}{c} G=HBD^{1/2}, \end{array}$$

where the elements of the matrix H are defined as $h_{mk}=e^{-j\left(m-1\right)\sin\left(\theta_{k}\right)}$, *m* is the antenna index, *k* is the device index, $\theta_{k}$ models the devices’ locations and is uniformly distributed in the interval [−π,π], the elements of the diagonal matrix B are defined as $b_{k}=e^{j\phi_{k}}$, $\phi_{k}$ is a uniformly distributed random variable defined in the range [−π,π] that models the phase shift associated with a random range between the array and the *k*-th device, and the elements of the diagonal matrix D that are defined as $d_{k}=\beta_{k}$ are the large-scale fading coefficients [1].

**Figure 1:**
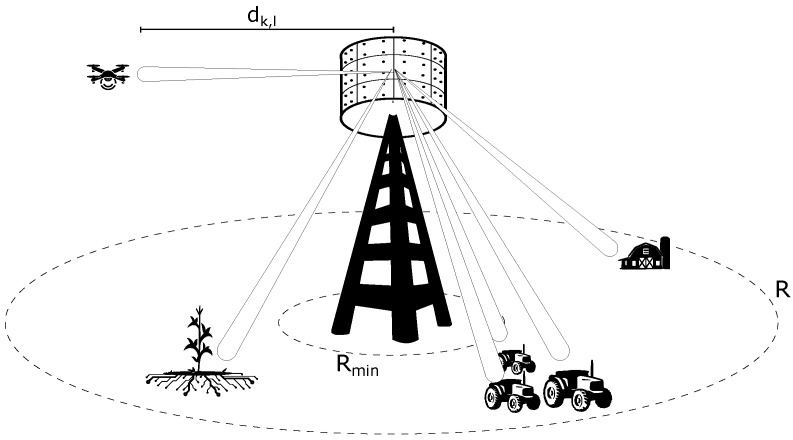
Illustration of the adopted system model.

The Friis’ free-space path-loss coefficients, $\beta_{km}$, are modeled as described in [30],

(2) $$\beta_{km}=\frac{\eta }{d_{k,m}^{2}},$$

where η is a constant equal to ${\left(\frac{\lambda }{4\pi }\right)}^{2}$ [31], $d_{k,m}$ is the distance between the *m*-th BS antenna and the *k*-th terminal’s antenna, and the path-loss exponent is equal to 2, which is the value used for free-space propagation. We consider isotropic antennas, i.e., the antennas have unitary gains (no directivity). As the distance between the *k*-th device and the antenna array is $d_{k,m}\gg \lambda$, then $d_{k,m}\approx d_{k}$, and consequently $\beta_{km}\approx \beta_{k}$, i.e., $\beta_{km}$ does not depend on the antenna index as the distance between the *k*-th device and the BS is much greater than the distance between the antennas. We consider that $d_{k}$ is a uniformly distributed random variable distributed in the interval $\left[R_{\min},R\right]$, where $R_{\min}$ is the minimum distance a device can be from the BS and *R* is the cell radius. The adopted system model is depicted in Figure 1.

## 4. Channel Sum-Capacity in LoS and Favorable Propagation Conditions

The distance between the device’s antennas and the BS’s antennas is not static as the devices are always moving within the cell (e.g., drones, cars, etc.). Therefore, the distance has to be treated as a random variable, resulting in a different channel realization for every time instant. As it is known [1], the instantaneous sum-capacity is not a meaningful performance metric under such random conditions. Here, we are interested in the capacity performance averaged over all different positions a device might be within the cell. Therefore, in order to assess the performance in this scenario, we need to employ the notion of ergodic capacity, which results in the following uplink sum link capacity [32]

(3) $$\begin{array}{cc} C & =E\left[\log_{2}\left|I_{M}+\rho GG^{H}\right|\right]\\ \; & =E\left[\log_{2}\left|I_{K}+\rho G^{H}G\right|\right], \end{array}$$

where ρ is the average SNR, also known as the average transmitted power of each device, and the expectation is taken over the joint distribution of all possible positions of the devices. As the additive white noise is assumed to have mean and variance equal to 0 and 1 respectively, therefore, ρ has, consequently, the interpretation of normalized transmit SNR and is therefore dimensionless [33]. It is important to highlight that the expectation in Equation (3) is taken in relation with devices’ channels, more specifically in relation to their positions within the cell (i.e., distances and angles of arrival), which are considered as random variables. The second line of Equation (3) is found by applying the Sylvester’s determinant theorem. Furthermore, we assume that the base station has perfect knowledge of the channel matrix G. The rationale behind the assumption of perfect knowledge of the channel matrix is that the results obtained with this assumption are readily comprehended and they bound the performance of massive MIMO systems.

Finding the exact ergodic capacity given by Equation (3) is quite a complex task that involves finding the distribution of the eigenvalues of $G^{H}G$ [34]. In this work, as we will show next, we are concerned with finding the sum-capacity considering a massive MIMO scenario and asymptotically favorable propagation [1]. In asymptotically favorable propagation scenarios, the channel vectors of different users become mutually orthogonal as the number of antennas, *M*, increases, i.e.,

(4) $$\frac{g_{i}^{H}g_{j}}{M}\;\;\;\overset{M\to \infty }{\to }\;\;\;0,\;\;\forall \;\;i\neq j.$$

The environment is said to offer asymptotically favorable propagation when Equation (4) is satisfied. The mutual orthogonality offered by environments exhibiting the asymptotic favorable propagation condition is the most beneficial situation from the perspective of sum-capacity maximization. Then, the sum-capacity in Equation (3) can be re-expressed as [1]

(5) $$\begin{array}{cc} C & =E\left[\log_{2}\left|I_{K}+\rho G^{H}G\right|\right]\\ \; & \leq E\left[\log_{2}\left(\prod_{k=1}^{K}{\left[I_{K}+\rho G^{H}G\right]}_{k,k}\right)\right]\\ \; & =E\left[\sum_{k=1}^{K}\log_{2}\left({\left[I_{K}+\rho G^{H}G\right]}_{k,k}\right)\right]\\ \; & =E\left[\sum_{k=1}^{K}\log_{2}\left(1+\rho ∥g_{k}{∥}^{2}\right)\right]. \end{array}$$

The inequality in the second line of Equation (5) is found applying the Hadamard inequality and assuming that $∥g_{k}{∥}^{2},\forall k$ is known, where $g_{k},\forall k$, are the columns of the channel matrix G. As will be shown later, this bound will be proven to be very tight. The equality in the second line of Equation (5) holds if and only if $G^{H}G$ is a diagonal matrix (i.e., the channel matrix G has mutually orthogonal columns) that must satisfy [35],

(6) $$g_{i}^{H}g_{j}=\left\{\begin{array}{cc} 0, & i,j=1,\ldots ,K,\mathrm{for}\;i\neq j\\ ∥g_{k}{∥}^{2}=M\beta_{k}, & \;\;k=1,\ldots ,K,\mathrm{for}\;i=j, \end{array}\right.$$

which is the case when the channel exhibits favorable propagation [36]. The equality $∥g_{k}{∥}^{2}=M\beta_{k}$ is detailed in Appendix A.

Given the assumption of channels exhibiting asymptotically favorable propagation, we know from [1] that the spatial signature vectors, denoted by $e^{j\phi_{k}}h_{k}$, become asymptotically mutually orthogonal and consequently, it can be shown that

(7) $$\frac{B^{H}H^{H}HB}{M}\;\;\;\overset{M\to \infty }{\approx }\;\;\;I_{K},$$

and therefore,

(8) $$\frac{G^{H}G}{M}=D^{1/2}\frac{B^{H}H^{H}HB}{M}D^{1/2}\;\;\;\overset{M\to \infty }{\approx }\;\;\;D,$$

hence, the results presented in this work hold for ULAs and URAs, as in the asymptotic regime of Equation (8) will always tend to the matrix containing the large-scale fading coefficients.

Now using $∥g_{k}{∥}^{2}=M\beta_{k}$ and $\beta_{k}=\frac{\eta }{d_{k}^{2}}$ in the last line of Equation (5), the sum-capacity upper bound can be rewritten as

(9) $$\begin{array}{c} C=\sum_{k=1}^{K}E\left[\log_{2}\left(1+\frac{\rho M\eta }{d_{k}^{2}}\right)\right]\\ =KE\left[\log_{2}\left(1+\frac{\rho M\eta }{d_{k}^{2}}\right)\right], \end{array}$$

where the last equality is due to the fact that $d_{k}$ is an i.i.d. random variable for all *k*.

Next, considering $z=\frac{1}{d_{k}^{2}}$ as a random variable denoted by *Z* with PDF given by

(10) $$\begin{array}{c} f_{Z}\left(z\right)=\left\{\begin{array}{cc} \frac{1}{2\left(R-R_{min}\right)z\sqrt{z}}, & \frac{1}{R^{2}}\leq z\leq \frac{1}{R_{min}^{2}},\\ 0, & \mathrm{otherwise}, \end{array}\right. \end{array}$$

then, Equation (9) can be re-expressed as

(11) $$\begin{array}{cc} C & =KE\left[\log_{2}\left(1+\frac{\rho M\eta }{d_{k}^{2}}\right)\right]\\ \; & =KE\left[\log_{2}\left(1+\rho M\eta z\right)\right]\\ \; & =\frac{K}{2(R-R_{min})}\int_{1/R^{2}}^{1/R_{min}^{2}}\log_{2}\left(1+\rho M\eta z\right)\frac{1}{z\sqrt{z}}dz, \end{array}$$

where the proof of Equation (10) is given in Appendix B. Next, solving Equation (11) with an integral solver [37], we find an exact closed-form expression given in Equation (12) for the capacity when the channel offers favorable propagation.

(12) $$C=\frac{K}{\left(R-R_{min}\right)}\frac{2\sqrt{\rho M\eta }\left(\tan^{-1}\left(\sqrt{\frac{\rho M\eta }{R_{min}^{2}}}\right)-\tan^{-1}\left(\sqrt{\frac{\rho M\eta }{R^{2}}}\right)\right)+R\log\left(\frac{\rho M\eta }{R^{2}}+1\right)-R_{min}\log\left(\frac{\rho M\eta }{R_{min}^{2}}+1\right)}{\log(2)}.$$

**Remark** **1.** *After analyzing Equation (12), we see that if we make the transmit power of each device equal to $P/M^{\alpha }$, where α>1, then the sum-capacity, C, will go to zero as M→∞. When α<1 the sum-capacity grows without bound as M→∞. This means that 1/M (i.e., α=1) is the fastest rate at which we can decrease the transmit power of each device and still have a fixed capacity as M→∞.*

Remark 1 clearly shows that as *M* grows without bound, the transmit power of each device can be reduced proportionally to 1/M and that the spectral efficiency increases by a factor of *K*, meaning that the BS can simultaneously serve *K* devices over the same time–frequency resources. This reduction of the transmit power per device is very important to power-constrained devices such as IoT devices.

### 4.1. Approximated Distribution of the Total Power Gain

The sum-capacity given by the first line of Equation (5) can be expressed in terms of the singular values $\left\{\sigma_{k}\right\}$ of the channel matrix G and, therefore, if $\sigma_{1}\geq \sigma_{2}\geq \cdots \sigma_{K}$ are the random ordered singular values of G, then Equation (5) can be rewritten as [1]

(13) $$\begin{array}{c} C=E\left[\sum_{k=1}^{K}\log_{2}\left(1+\rho \sigma_{k}^{2}\right)\right]. \end{array}$$

By comparing Equations (5) and (13), it is possible to conclude that

(14) $$\sigma_{k}^{2}={∥g_{k}∥}^{2}=M\beta_{k}.$$

The singular values represent the channel gains of the parallel channels after the singular value decomposition (SVD) of the system [32]. Next we present the squared Frobenius norm of G when the environment offers favorable propagation, and consequently, G is full rank

(15) $${∥G∥}_{F}^{2}=\sum_{m=1}^{M}\sum_{k=1}^{K}{\left|g_{mk}\right|}^{2}=Tr\left(G^{H}G\right)=\sum_{k=1}^{K}\sigma_{k}^{2}.$$

The last term of Equation (15) can be interpreted as the total power gain of the channel matrix if one spreads the energy equally between all the antennas [32]. Therefore, comparing Equations (14) and (15), we clearly see that the singular values have the same distribution as the Friis’ free-space path-loss coefficients, $\beta_{k},\forall k$. The summation of the *K* free-space path-loss coefficients, is a random variable that can be approximated by a Gamma distribution as shown in Appendix D.

### 4.2. A Lower-Bound for the Sum-Capacity

First we define the following Jensen’s inequality [38]

(16) $$\begin{array}{c} E\left[\log_{2}\left(1+z\right)\right]\geq \log_{2}\left(1+\frac{1}{E\left[\frac{1}{z}\right]}\right), \end{array}$$

where $z=\frac{1}{u}$ for z>0. Therefore, Equation (9) can be rewritten as

(17) $$\begin{array}{cc} C & =KE\left[\log_{2}\left(1+\frac{\rho M\eta }{d_{k}^{2}}\right)\right]\\ \; & \geq K\log_{2}\left(1+\frac{\rho M\eta }{E\left[d_{k}^{2}\right]}\right). \end{array}$$

Remembering that $f_{d}\left(r\right)=\frac{1}{R-R_{\min}},\;R_{\min}\leq r\leq R$, then

(18) $$\begin{array}{c} E\left[d_{k}^{2}\right]=\frac{R^{3}-R_{min}^{3}}{3(R-R_{min})}. \end{array}$$

For proof of Equation (18), see Appendix C. Therefore, Equation (17) can be re-expressed as

(19) $$\begin{array}{c} C\geq K\log_{2}\left(1+\frac{3\rho M\eta (R-R_{min})}{R^{3}-R_{min}^{3}}\right). \end{array}$$

### 4.3. An Upper-Bound for the Sum-Capacity

Again, we can use the Jensen’s inequality as [1]

(20) $$\begin{array}{c} E\left[\log_{2}\left(1+z\right)\right]\leq \log_{2}\left(1+E\left[z\right]\right), \end{array}$$

for z>0. Therefore, Equation (9) can be rewritten as

(21) $$\begin{array}{cc} C & =KE\left[\log_{2}\left(1+\frac{\rho M\eta }{d_{k}^{2}}\right)\right]\\ \; & \leq K\log_{2}\left(1+\rho M\eta E\left[\frac{1}{d_{k}^{2}}\right]\right). \end{array}$$

Remembering that $f_{d}\left(r\right)=\frac{1}{R-R_{\min}},\;R_{\min}\leq r\leq R$, then

(22) $$\begin{array}{c} E\left[\frac{1}{d_{k}^{2}}\right]=\frac{1}{RR_{min}}. \end{array}$$

For proof of Equation (22), see Appendix C. Therefore, Equation (21) can be re-expressed as

(23) $$\begin{array}{c} C\leq K\log_{2}\left(1+\frac{\rho M\eta }{RR_{min}}\right). \end{array}$$

### 4.4. Low-SNR Regime

For the low-SNR regime, we have the following approximation: $\log_{2}\left(1+x\right)\approx x\log_{2}\left(e\right)$, when x≪1, then Equation (9) can be expressed as

(24) $$\begin{array}{cc} C & \approx KE\left[\frac{\rho M\eta }{d_{k}^{2}}\log_{2}\left(e\right)\right]\\ \; & =K\rho M\eta \log_{2}\left(e\right)E\left[\frac{1}{d_{k}^{2}}\right]\\ \; & =\frac{K\rho M\eta \log_{2}\left(e\right)}{RR_{min}}. \end{array}$$

In Equation (24), we see that the sum-capacity linearly increases with the average SNR, ρ, and/or with the number of antennas, *M*.

### 4.5. High-SNR Regime

For the high-SNR regime, we have the following approximation: $\log_{2}\left(1+x\right)\approx \log_{2}\left(x\right)$, when x≫1, then Equation (9) can be expressed as

(25) $$\begin{array}{cc} C & \approx KE\left[\log_{2}\left(\frac{\rho M\eta }{d_{k}^{2}}\right)\right]\\ \; & =\frac{K}{2(R-R_{min})}\int_{1/R^{2}}^{1/R_{min}^{2}}\log_{2}\left(\rho M\eta z\right)\frac{1}{z\sqrt{z}}dz, \end{array}$$

where $z=\frac{1}{d_{k}^{2}}$. Solving Equation (25) with an integral solver [37], we find an exact closed-form expression for the capacity when the channel offers favorable propagation in the high-SNR regime. The exact closed-form expression for the approximated sum-capacity in Equation (25) is given by Equation (26).

(26) $$\begin{array}{c} C\approx \frac{K}{\left(R-R_{min}\right)}\frac{\log(\rho M\eta )\;(R-R_{min})+2\left[R-R_{min}-R\log\left(R\right)+R_{min}\log(R_{min})\right]}{\log(2)}. \end{array}$$

In Equation (26), differently from Equation (24), we see that the sum-capacity logarithmically increases with the average SNR, ρ, and/or with the number of antennas, *M*. Therefore, in the high-SNR regime, an increase in the transmit power and/or in the number of antennas, *M*, is much less impressive than in the low-SNR regime case.

### 4.6. Instantaneous Sum-Capacity PDF in Low-SNR Regime and Favorable Propagation Condition

The instantaneous sum-capacity in the low-SNR regime and favorable propagation condition is given by

(27) $$\begin{array}{cc} C_{\mathrm{inst}.} & =\sum_{k=1}^{K}\log_{2}\left(1+\frac{\rho M\eta }{d_{k}^{2}}\right)\\ \;\;\;\; & \overset{\frac{\rho M\eta }{d_{k}^{2}}\ll 1}{\approx }\;\;\rho M\eta \log_{2}\left(e\right)\sum_{k=1}^{K}\frac{1}{d_{k}^{2}}, \end{array}$$

which is a random variable that depends on the distance of the *k*-th device to the BS, denoted by $d_{k}$.

**Lemma** **1.** *By empirically comparing the normalized histogram of the random variable given by Equation (27) against the theoretical PDF of a Gamma random variable we notice that as K increases that $C_{\mathrm{inst}.}$ can be approximated by the Gamma PDF with parameters κ and θ given by*

(28) $$\kappa =\frac{3KRR_{min}}{{\left(R-R_{min}\right)}^{2}},$$

(29) $$\theta =\frac{\rho M\eta \log_{2}\left(e\right){\left(R-R_{min}\right)}^{2}}{3R^{2}R_{min}^{2}}.$$

*This comparison is shown in Section 6. The parameters κ and θ are found following the same rationale used in Appendix D.*

### 4.7. Instantaneous Sum-Capacity PDF In High-SNR Regime and Favorable Propagation Condition

The instantaneous sum-capacity in the high-SNR regime and favorable propagation condition is given by

(30) $$\begin{array}{cc} C_{\mathrm{inst}.} & =\sum_{k=1}^{K}\log_{2}\left(1+\frac{\rho M\eta }{d_{k}^{2}}\right)\\ \;\;\;\; & \overset{\frac{\rho M\eta }{d_{k}^{2}}\gg 1}{\approx }\;\;\sum_{k=1}^{K}\log_{2}\left(\frac{\rho M\eta }{d_{k}^{2}}\right), \end{array}$$

which is a random variable that depends on the distance of the *k*-th device to the BS, denoted by $d_{k}$.

**Lemma** **2.** *By empirically comparing the normalized histogram of the random variable given by Equation (30) against the theoretical PDF of a Gamma random variable we notice that as M increases that $C_{\mathrm{inst}.}$ can be approximated by the Gamma PDF with parameters κ and θ given by Equations (31) and (32), respectively, where a=log(ρMη), $b=\log\left(\frac{\rho M\eta }{R_{\min}^{2}}\right)$, $c=\log\left(\frac{\rho M\eta }{R^{2}}\right)$, $d=\log\left(\frac{1}{R_{\min}^{2}}\right)$, and $e=\log\left(\frac{1}{R^{2}}\right)$. This comparison is shown in Section 6. The parameters κ and θ are found as described in Appendix E.*

(31) $$\begin{array}{c} \kappa =\frac{\frac{K}{\left(R-R_{\min}\right)}{\left(\frac{b+2}{R}-\frac{c+2}{R_{\min}}\right)}^{2}}{\frac{1}{RR_{\min}}\left(\frac{2e\left(a+2\right)+e^{2}+a\;\left(a+4\right)+8}{R_{\min}}-\frac{2d\left(a+2\right)+d^{2}+a\left(a+4\right)+8}{R}\right)-\frac{1}{\left(R-R_{\min}\right)}{\left(\frac{b+2}{R}-\frac{c+2}{R_{\min}}\right)}^{2}}. \end{array}$$

(32) $$\begin{array}{c} \theta =\frac{\frac{RR_{\min}}{\left(R-R_{\min}\right)}{\left(\frac{b+2}{R}-\frac{c+2}{R_{\min}}\right)}^{2}-\frac{2e\left(a+2\right)+e^{2}+a\;\left(a+4\right)+8}{R_{\min}}+\frac{2d\left(a+2\right)+d^{2}+a\left(a+4\right)+8}{R}}{\log\left(2\right)\left(\frac{b+2}{R}-\frac{c+2}{R_{\min}}\right)}. \end{array}$$

### 4.8. Outage Probability

Therefore, based on the knowledge of the approximated PDFs, it is possible to use the Gamma’s cumulative density function (CDF) as a way to analyze the outage probability, $C_{\mathrm{out}}$, in low- and high-SNR regimes. Results comparing the PDF and CDF of the actual random variable and those of a Gamma random variable are presented and discussed in Section 6. Outage probability is the probability that a certain sum-capacity cannot be reached and is defined as

(33) $$\begin{array}{ccc} P_{\mathrm{out}.} & = & \mathrm{Pr}\{C_{\mathrm{inst}.}\leq C_{\mathrm{out}.}\} \end{array}$$

(34) $$\begin{array}{cc} \;\;\;\overset{M\to \infty }{\approx }\;\;\; & \frac{1}{\Gamma (\kappa )}\gamma \left(\kappa ,\frac{C_{\mathrm{out}.}}{\theta }\right), \end{array}$$

where Γ(.) is the gamma function, γ(.,.) is the incomplete gamma function, and κ and θ are given by Equations (28) and (29) for the low-SNR regime and by Equations (31) and (32) for the high-SNR regime, respectively.

## 5. Average Distance from Favorable Propagation

The favorable propagation is an important metric which is defined as mutual orthogonality among the vector-valued channels to the terminals [26]. The measure is one of the key properties of the radio channel that is exploited in massive MIMO. From [26], we can further characterize favorable propagation as

(35) $$\Delta_{C}=\frac{KE\left[\log_{2}\left(1+\rho M\beta_{k}\right)\right]-E\left[\log_{2}\left|I_{K}+\rho G^{H}G\right|\right]}{E\left[{log}_{2}\left|I_{K}+\rho G^{H}G\right|\right]}.$$

As can be seen from Equation (35), when $\Delta_{C}=0$, the channel offers favorable propagation. The measure defined in Equation (35) is an extension of the one presented in [1] for the ergodic capacity case assumed in this work.

## 6. Simulation Results and Discussion

In this section, we present the results of several simulations that were designed to assess the findings we have reported in this work. Initially, we assess the tightness of the derived exact and lower/upper bounds for the sum-capacity in favorable propagation against the actual capacity as the number of antennas grows. Next, we evaluate the tightness of the derived capacity equations over the variation of the average SNR. Then, we show that the sum-capacity for low- and high-SNR regimes can be accurately described by the derived approximated closed-form equations. In the sequence, we demonstrate the power scaling law for different values of α and show that the transmit power can be reduced as the number of antennas grows. Next, we prove that the total power gain of the channel matrix can be approximated by a Gamma distribution. To conclude, the remaining simulation results present approximations for distribution of the instantaneous sum-capacity in low- and high-SNR regimes and show that by employing those distributions, the outage probabilities can be accurately calculated for these two cases as the number of devices and antennas grows, respectively. All results presented here have the following simulation setup parameters R=100 [m], $R_{min}=10$ [m], and λ=0.01070687 [m], which is equivalent to a carrier frequency of 28 GHz.

**Figure 2:**
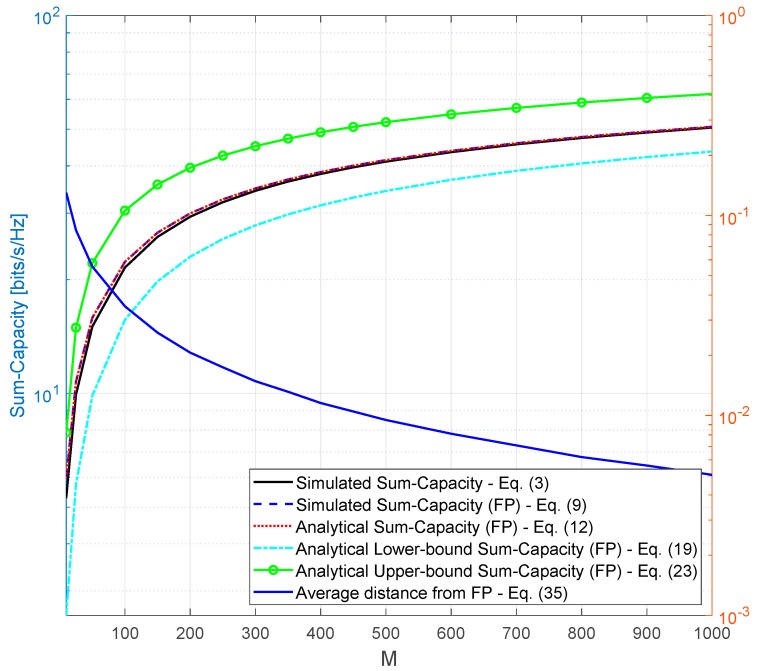
Simulation considering K=10, ρ=80 [dB], R=100 [m], $R_{min}=10$ [m], and λ=0.01070687 [m].

Figure 2 has the following simulation setup parameters K=10 and ρ=80 [dB]. As can be seen, the simulated capacity is within the lower and upper bound ranges as expected. It is also possible to see that the simulated and analytical capacities in favorable propagation match each other, showing that the derived analytical closed-form is tight for the favorable propagation case. Moreover, the simulated capacity asymptotically approaches the capacity in favorable propagation as the number of antennas, *M*, grows, proving that favorable propagation is asymptotically achieved as *M* grows. It is also important to highlight that the analytical capacity in favorable propagation, given by Equation (12), provides a good approximation for the capacity. For example, for M=100 the simulated capacity is equal to 21.53 bits/s/Hz and the analytical capacity in favorable propagation is equal to 22.3 bits/s/Hz.

Figure 2 also presents on the right x-axis the average distance from favorable propagation as defined in Equation (35). As can be noticed, the average distance asymptotically decreases as the number of antennas grows, starting at 0.13 for M=10 and decreasing to 0.005049 for M=1000. This result is another indication that favorable propagation is asymptotically achieved as *M* grows.

**Figure 3:**
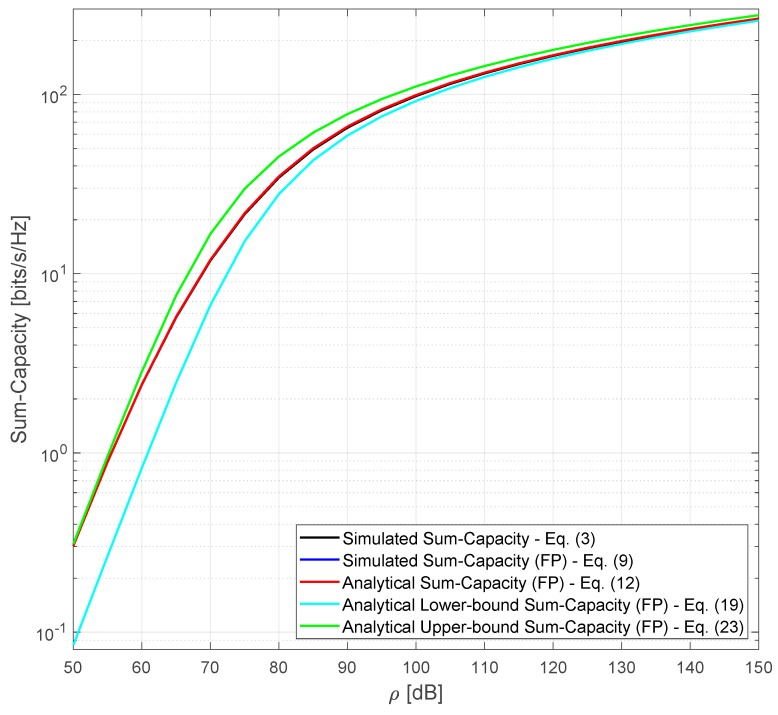
Simulated and analytical sum-capacity considering M=300.

Figure 3 compares the sum-capacity over the variation of the average SNR, ρ, for the same simulation parameters used for the results in Figure 2 and *M* constant and equal to 300 antennas. As expected, the sum-capacity, simulated and analytical, stays within the lower and upper capacity bounds. For low-SNR values the sum-capacity is closer to the upper bound and as the SNR increases, we see that both lower and upper bounds converge to the sum-capacity.

**Figure 4:**
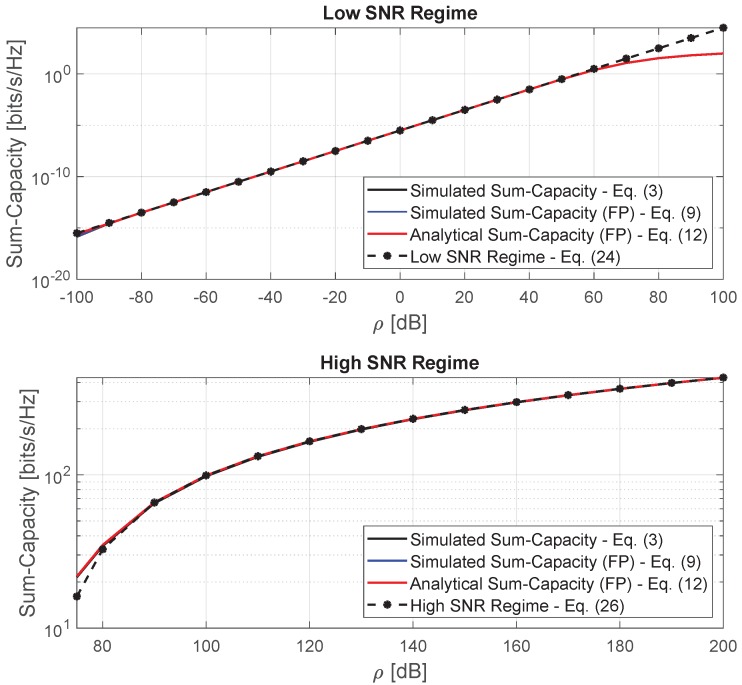
Sum-capacity for low- and high-SNR regimes versus average signal-to-interference ratio, ρ.

In Figure 4 we present the results of the sum-capacity for low- and high-SNR regimes in favorable propagation condition versus the average signal-to-interference ratio, ρ, with *M* constant and equal to 300 antennas. As can be seen, Equations (24) and (26) represent the sum-capacity fairly well for the low- and high-SNR regimes, respectively. In the upper part of the figure, we show the sum-capacity for the low-SNR regime and it is possible to see that the approximated expression given by Equation (24) closely follows the sum-capacity until around 60 [dB]. In the lower part of the figure, we show the sum-capacity for the high-SNR regime and we also see that the approximated expression given by Equation (26) closely follows the sum-capacity for SNR values greater than 80 [dB]. The figure also shows that the sum-capacity grows linearly and logarithmically with the average SNR for the low- and high-SNR regimes, respectively, confirming what was discussed in Section 4.4 and Section 4.5.

**Figure 5:**
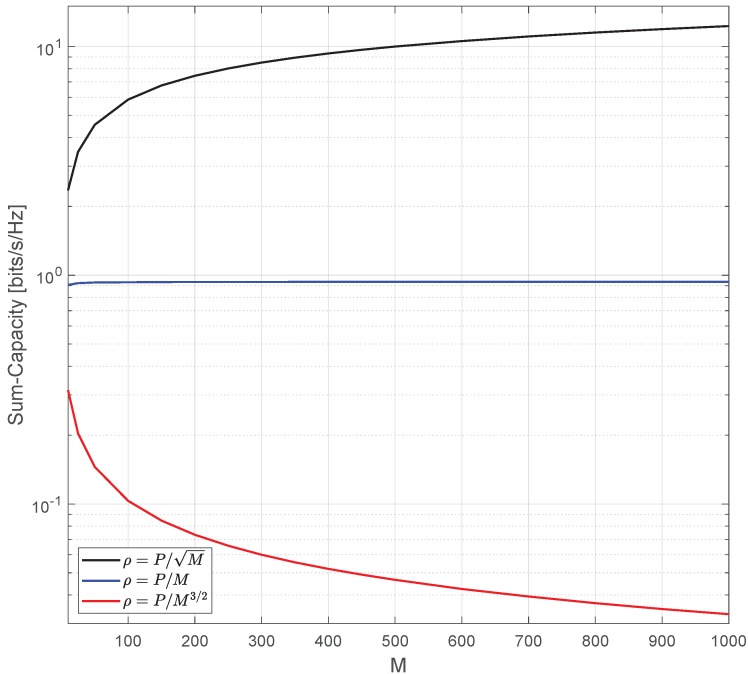
Demonstration of the power scaling law for different α values.

Figure 5 shows how the spectral efficiency behaves as $\rho =P/M^{\alpha }$, where α=1/2,1 and 3/2, respectively. The following simulation setup parameters were used: K=10 and P=80 [dB]. As expected and stated in Remark 1, when α=1 and *M* increases, the capacity becomes constant no matter the number of antennas. However, when α=1/2 the capacity grows logarithmically fast with *M* when M→∞ and tends to zero when α=3/2 and M→∞. These results attest that the transmit power of each device can be reduced proportionally to *M*.

**Figure 6:**
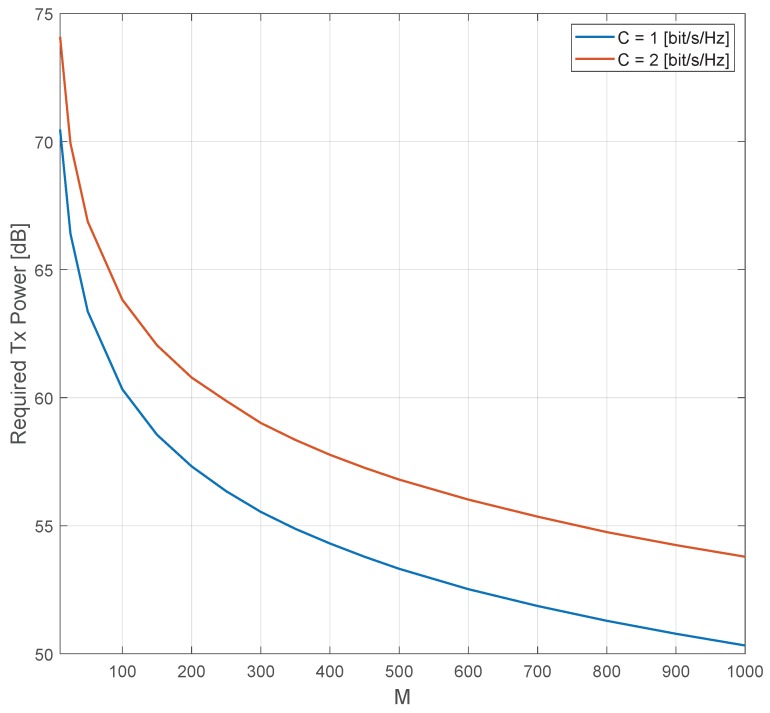
Required transmit power to achieve 1 and 2 bits/s/Hz as a function of the number of antennas.

In Figure 6, we show the required transmit power per device that is needed to achieve fixed capacities of 1 and 2 bits/s/Hz, respectively. As expected and predicted by Remark 1, the transmit power can be reduced by approximately 3 [dB] by doubling the number of antennas, *M*, for both cases, i.e., 1 and 2 bits/s/Hz.

**Figure 7:**
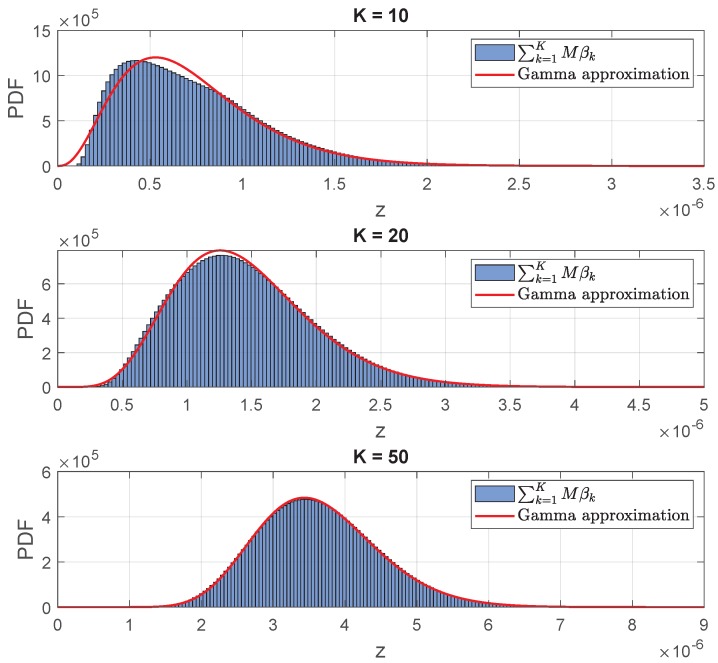
Gamma probability density function (PDF) for number of devices equal to K=10, 20, and 50 respectively.

Figure 7 shows the comparison between the normalized histogram of the random variable *Z*, where *Z* is defined in Lemma A1, and the Gamma PDF for number of devices equal to K=10, 20, and 50, respectively. These results were obtained with M=100. The histogram, shown in blue, is the histogram of the random variable $Z=\sum_{k=1}^{K}M\beta_{k}$. As can be seen, the histogram approaches the Gamma PDF as the number of devices increases.

**Figure 8:**
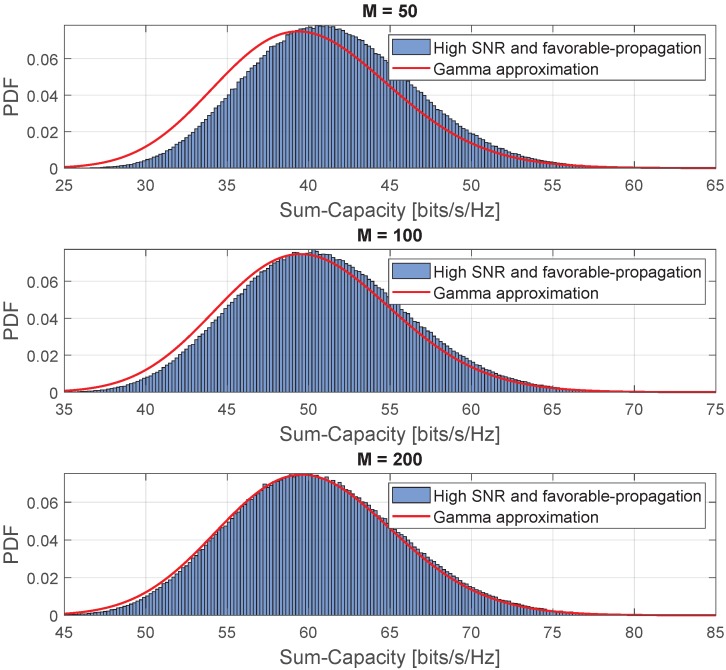
Comparison of the approximated PDF for the instantaneous sum-capacity in the high-SNR regime and favorable propagation condition.

Figure 8 shows the comparison of the instantaneous sum-capacity in the high-SNR regime and favorable propagation condition and its approximation with the Gamma PDF. This comparison was obtained with the following parameters K=10 and ρ=90 [dB]. As can be seen, for the high SNR, the Gamma PDF fits the PDF of the instantaneous sum-capacity random variable as the number of antennas grows.

**Figure 9:**
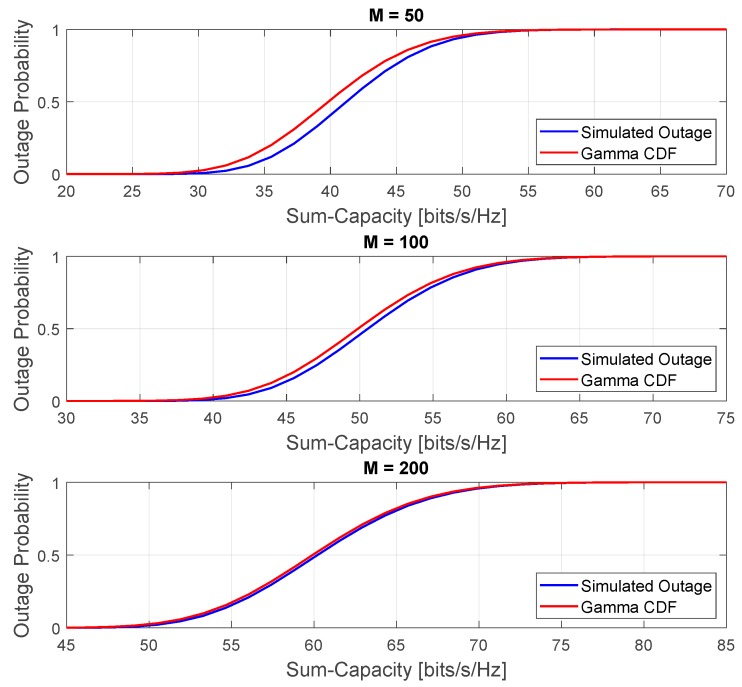
Comparison between the approximated and simulated outage probabilities in the high-SNR regime and favorable propagation condition.

Figure 9 shows the comparison between the approximated and simulated outage probabilities in the high-SNR regime and favorable propagation condition. The simulation parameters are the same used for generating the results in Figure 8. As can also be seen, for the high SNR, the Gamma CDF fits the simulated outage probability of the instantaneous sum-capacity random variable as the number of antennas grows. Additionally, we can also notice that the probability of achieving higher capacities increases with the number of antennas. The increased probability of higher capacity is due to the asymptotic favorable propagation offered by the channel as the number of antennas grows, i.e., the channels become asymptotically orthogonal as the number of antennas increases and consequently, the cross-talk interference is asymptotically mitigated.

**Figure 10:**
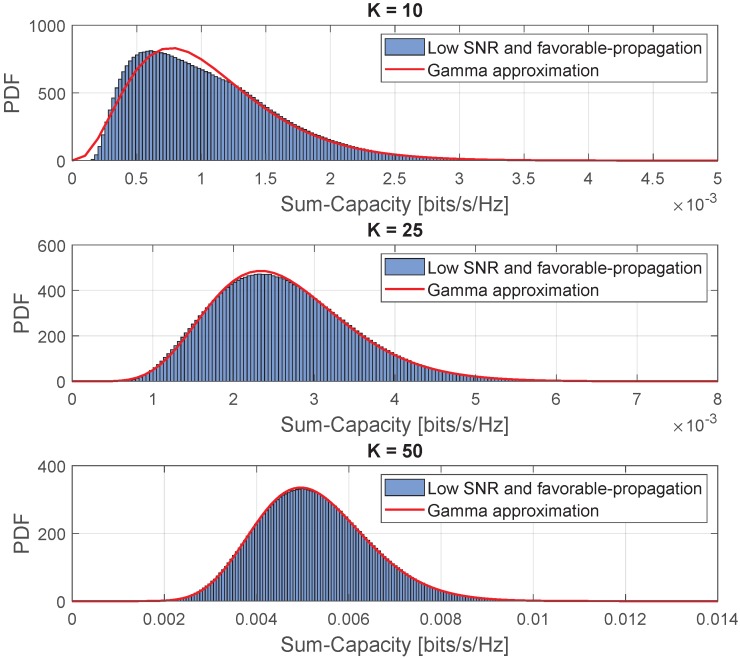
Comparison of the approximated PDF for the instantaneous sum-capacity in the low-SNR regime and favorable propagation condition.

Figure 10 shows the comparison of the instantaneous sum-capacity in the low-SNR regime and favorable propagation condition and its approximation with the Gamma PDF. This comparison was obtained with the following parameters M=100 and ρ=30 [dB]. As can be seen, for the low SNR, the Gamma PDF fits the PDF of the instantaneous sum-capacity random variable as the number of devices simultaneously served through the same time–frequency resources grows.

**Figure 11:**
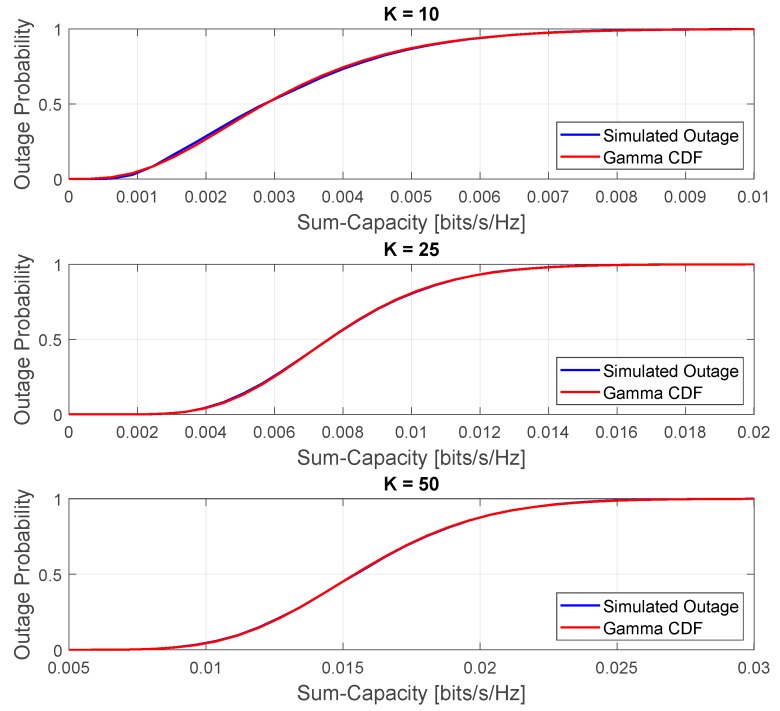
Comparison between the approximated and simulated outage probabilities in the low-SNR regime and favorable propagation condition.

Figure 11 shows the comparison between the approximated and simulated outage probabilities in the low-SNR regime and favorable propagation condition. This comparison was obtained with the following parameters M=300 and ρ=30 [dB]. As can also be seen, for the low SNR, the Gamma CDF fits the simulated outage probability of the instantaneous sum-capacity random variable as the number of served devices grows.

Although we consider a carrier frequency of 28 GHz in our analysis, our derived results can be applied to any carrier frequency under the assumptions of favorable propagation, a dominant LoS channel with no multi-path fading and devices randomly moving around the cell. Under the aforementioned assumptions, the channel vectors of different devices become mutually orthogonal, resulting in (8), which is a diagonal matrix with the free-space path-loss coefficients in its main diagonal. In (2), we see that the free-space path-loss coefficients are directly proportional to the η parameter, which takes the wavelength of the carrier frequency into account. The attenuation caused by the path-loss is mitigated by adjusting (i.e., increasing) the device’s transmission power or number of antennas. Therefore, considering the aforementioned assumptions, the analysis we provide in this work can be straightforwardly extended to other carrier frequencies given that the device’s Tx power or the number of antennas (the greater the number of antennas the higher is the harnessed energy, i.e., received energy) is also adjusted to combat the path-loss.

**Figure 12:**
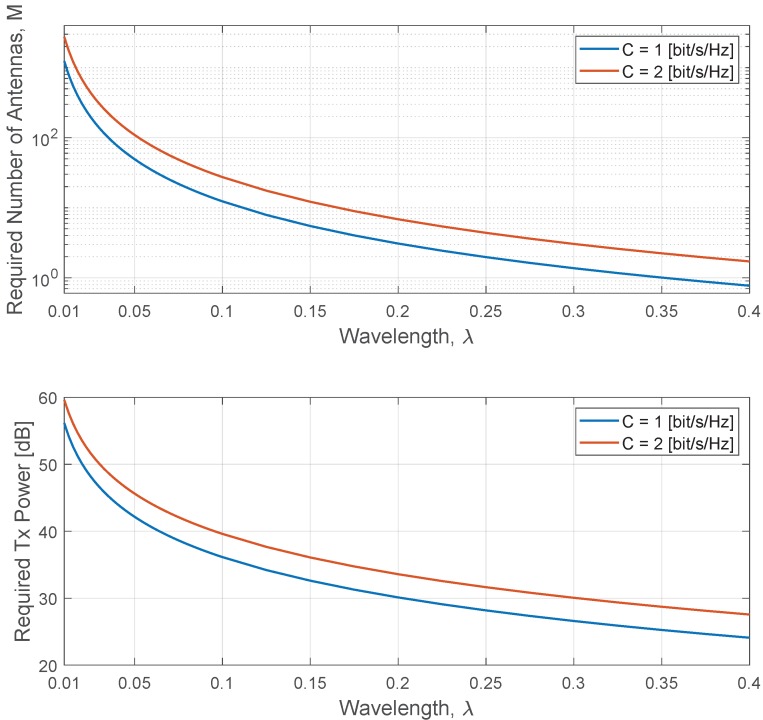
Required number of antennas and Tx power versus the wavelength, λ.

Figure 12 presents in its upper part the required number of antennas necessary to achieve sum-capacities of 1 and 2 bits/s/Hz, respectively, for a variation of the carrier frequency, which is represented by the wavelength, λ, and with a fixed power ρ=50 dB. The lower part of the figure shows the required Tx power necessary to achieve sum-capacities of 1 and 2 bits/s/Hz, respectively, for a variation of the carrier frequency and with a fixed number of antennas M=300. As can be seen, the required number of antennas and Tx power decreases as the λ increases. It is also interesting to mention that both required values decrease by 4 (or by 6 dB for the required Tx power case) as λ increases. The decrease rate of 4 is due to the fact that the path-loss exponent is equal to 2 (see, (2)), which doubles any increase of λ, i.e., the carrier frequency. Therefore, the number of antennas should be increased by 4 or the Tx power by 6 dB if one wants to double the carrier frequency and keep the same sum-rate capacity for the same constant parameters (i.e., ρ and *M*, respectively). This shows that the same capacity can be kept as long as the number of antennas or Tx power is adjusted to combat the free-space path-loss.

## 7. Conclusions

In this work, we investigated ways to find capacity limits concerning the number of users, number of BS antennas, and SNR. By assuming a dominant LoS environment in a massive MIMO scenario, it was possible to derive analytical expressions for the channel capacity. Convenient simplifications on expressions were possible working at low- and high-SNR regimes. Furthermore, it is demonstrated that the Gamma PDF can approximate the PDF of the instantaneous channel sum-capacity as the number of antennas grows for both cases of low- and high-SNR regimes. A second important demonstration is that a Gamma PDF can also approximate the PDF of the summation of the channel’s singular values as the number of devices increases. Finally, the utility of such a framework is useful for a massive number of IoT devices as we show that the transmit power of each device can be made inversely proportional to the number of BS antennas.

## Appendix A. Derivation of $∥g_{k}{∥}^{2}=M\beta_{k}$

From Equation (1), we can write each element $g_{k,m}$ of $g_{k}$ as

(A1) $$g_{k,m}=\sqrt{\beta_{k}}e^{j\phi_{k}}e^{-j\left(m-1\right)\sin\left(\theta_{k}\right)}.$$

Therefore, using the very definition of the product of two matrices [39], we have

(A2) $$\begin{array}{cc} {∥g_{k}∥}^{2}\; & =\sum_{m=1}^{M}{\left|\sqrt{\beta_{k}}e^{j\phi_{k}}e^{-j\left(m-1\right)\sin\left(\theta_{k}\right)}\right|}^{2}\\ \; & =\sum_{m=1}^{M}{\left|\sqrt{\beta_{k}}\right|}^{2}{\left|e^{j\phi_{k}}\right|}^{2}{\left|e^{-j\left(m-1\right)\sin\left(\theta_{k}\right)}\right|}^{2}, \end{array}$$

where ${\left|e^{j\phi_{k}}\right|}^{2}={\left|e^{-j\left(m-1\right)\sin\left(\theta_{k}\right)}\right|}^{2}=1$ and ${\left|\sqrt{\beta_{k}}\right|}^{2}=\beta_{k}$, therefore

(A3) $${∥g_{k}∥}^{2}=\sum_{m=1}^{M}\beta_{k}=M\beta_{k}.$$

## Appendix B. Derivation of the PDF of the Distance Random Variable

Given that the PDF of *Y* is defined as

(A4) $$f_{Y}\left(y\right)=\frac{1}{R-R_{min}},\;\;\;R_{min}\leq y\leq R,$$

and the strictly monotonic differentiable function $Z=g\left(Y\right)=1/Y^{2}$, then the PDF of *Z* is given by

(A5) $$f_{Z}\left(z\right)=\frac{f_{Y}\left(g^{-1}\left(Z\right)\right)}{|g^{\prime }\left(Y\right)|},$$

where $g^{\prime }\left(Y\right)=-2/Y^{3}$ and $g^{-1}\left(Z\right)=1/\sqrt{Z}$. Therefore,

(A6) $$\begin{array}{cc} f_{Z}\left(z\right) & =\frac{f_{Y}\left(g^{-1}\left(Z\right)\right)}{|g^{\prime }\left(Y\right)|}=\frac{\frac{1}{R-R_{min}}}{|\frac{-2}{y^{3}}|}\\ \; & =\frac{1}{2\left(R-R_{min}\right)z\sqrt{z}}, \end{array}$$

where in the last equality we used the fact that $y=1/\sqrt{z}$.

## Appendix C. Useful Results

Calculation of $E\left[d_{k}^{2}\right]$

(A7) $$\begin{array}{c} E\left[d_{k}^{2}\right]=\int_{R_{\min}}^{R}r^{2}f_{d}\left(r\right)dr=\int_{R_{\min}}^{R}\frac{r^{2}}{R-R_{\min}}dr=\frac{R^{3}-R_{min}^{3}}{3(R-R_{min})}. \end{array}$$

Calculation of $E\left[\frac{1}{d_{k}^{2}}\right]$

(A8) $$\begin{array}{c} E\left[\frac{1}{d_{k}^{2}}\right]=\int_{R_{\min}}^{R}\frac{1}{r^{2}}f_{d}\left(r\right)dr=\int_{R_{\min}}^{R}\frac{1}{r^{2}\left(R-R_{\min}\right)}dr=\frac{1}{RR_{min}}. \end{array}$$

Calculation of $E\left[\frac{1}{d_{k}^{4}}\right]$

(A9) $$\begin{array}{c} E\left[\frac{1}{d_{k}^{4}}\right]=\int_{R_{\min}}^{R}\frac{1}{r^{4}}f_{d}\left(r\right)dr=\int_{R_{\min}}^{R}\frac{1}{r^{4}\left(R-R_{\min}\right)}dr=\frac{R^{2}+RR_{min}+R_{min}^{2}}{3R^{3}R_{min}^{3}}. \end{array}$$

Calculation of $E\left[\log_{2}\left(\frac{c}{d_{k}^{2}}\right)\right]$

(A10) $$\begin{array}{cc} E\left[\log_{2}\left(\frac{c}{d_{k}^{2}}\right)\right] & =\int_{R_{min}}^{R}\log_{2}\left(\frac{c}{r^{2}}\right)\frac{1}{R-R_{min}}dr\\ \; & =\frac{\log\left(c\right)+\frac{2(R-R_{min}-R\log\left(R\right)+R_{min}\log\left(R_{min}\right))}{R-R_{min}}}{\log(2)} \end{array}$$

## Appendix D. Approximated Distribution of the Total Power Gain of the Channel Matrix

**Lemma** **A1.**
*Let $\beta_{k}=\eta /d_{k}^{2}$, where $d_{k},\forall k$ are i.i.d. random variables following the uniform distribution with PDF given by $1/(R-R_{min})$, therefore, as the number of served devices increases, the PDF of $Z=\sum_{k=1}^{K}M\beta_{k}$ can be approximated by a Gamma distribution with parameters $\kappa =\frac{3KRR_{min}}{{\left(R-R_{min}\right)}^{2}}$ and $\theta =\frac{M\eta {\left(R-R_{min}\right)}^{2}}{3R^{2}R_{min}^{2}}$, i.e., Γ(κ,θ).*

**Proof.** This is empirically proven by comparing the normalized histogram of *Z* against the theoretical PDF of a Gamma random variable with the parameters defined earlier. The parameters κ and θ are found by using the mean and variance statistics of *Z*, where $\kappa =\frac{\mu_{Z}^{2}}{\sigma_{Z}^{2}}$ and $\theta =\frac{\sigma_{Z}^{2}}{\mu_{Z}}$. These relations are derived based on the definitions of mean and variance of a Gamma random variable. The mean of *Z* is given by

(A11) $$\begin{array}{cc} \mu_{Z} & =M\eta \sum_{k=1}^{K}E\left[\frac{1}{d_{k}^{2}}\right]\\ \; & =MK\eta E\left[\frac{1}{d_{k}^{2}}\right]\\ \; & =\frac{MK\eta }{RR_{min}}, \end{array}$$

where we used the assumption that $d_{k},\forall k$ are i.i.d. random variables and the results in Appendix C. Next, the variance of *Z* is given by

(A12) $$\begin{array}{c} \sigma_{Z}^{2}=E\left[Z^{2}\right]-\mu_{Z}^{2}, \end{array}$$

where

(A13) $$\begin{array}{cc} E[Z^{2}] & =M^{2}K\left\{E\left[\beta^{2}\right]+\left(K-1\right)E{\left[\beta_{k}\right]}^{2}\right\}\\ \; & =M^{2}\eta^{2}K\left\{E\left[\frac{1}{d_{k}^{4}}\right]+\left(K-1\right)E{\left[\frac{1}{d_{k}^{2}}\right]}^{2}\right\}\\ \; & =M^{2}\eta^{2}K\left[\frac{R^{2}+RR_{min}+R_{min}^{2}}{3R^{3}R_{min}^{3}}-\frac{\left(K-1\right)}{R^{2}R_{min}^{2}}\right], \end{array}$$

where $E\left[\frac{1}{d_{k}^{4}}\right]$ is calculated in Appendix C. Therefore, plugging Equation (A13) into Equation (A12) we have

(A14) $$\begin{array}{c} \sigma_{Z}^{2}=\frac{K\eta^{2}M^{2}{\left(R-R_{min}\right)}^{2}}{3R^{3}R_{min}^{3}}. \end{array}$$

The proof is concluded by replacing Equations (A11) and (A14) into the definitions of κ and θ. ☐

## Appendix E. Approximated Distribution of the Instantaneous Sum-Capacity in the High-SNR Regime and Favorable Propagation

In this appendix we demonstrate how the parameters κ and θ are derived. These parameters, which are used to approximate the instantaneous sum-capacity PDF in the high-SNR regime and favorable propagation, are found by using the mean and variance statistics of the random variable given by Equation (30), where $\kappa =\frac{\mu_{C_{\mathrm{inst}.}}^{2}}{\sigma_{C_{\mathrm{inst}.}}^{2}}$ and $\theta =\frac{\sigma_{C_{\mathrm{inst}.}}^{2}}{\mu_{C_{\mathrm{inst}.}}}$. These relations are derived based on the definitions of mean and variance of a Gamma random variable. The mean of Equation (30) is given by

(A15) $$\begin{array}{cc} \mu_{C_{\mathrm{inst}.}} & =E[C_{\mathrm{inst}.}]\\ \; & =E\left[\sum_{k=1}^{K}\log_{2}\left(\frac{\rho M\eta }{d_{k}^{2}}\right)\right]\\ \; & =\sum_{k=1}^{K}E\left[\log_{2}\left(\frac{\rho M\eta }{d_{k}^{2}}\right)\right]\\ \; & =KE\left[\log_{2}\left(\frac{\rho M\eta }{d_{k}^{2}}\right)\right]\\ \; & =K\frac{\log\left(\rho M\eta \right)+\frac{2(R-R_{min}-R\log\left(R\right)+R_{min}\log\left(R_{min}\right))}{R-R_{min}}}{\log(2)}, \end{array}$$

where we used the assumption that $d_{k},\forall k$ are i.i.d. random variables and the result in Appendix C. Next, the variance of Equation (30) is given by

(A16) $$\begin{array}{c} \sigma_{C_{\mathrm{inst}.}}^{2}=E\left[C_{\mathrm{inst}.}^{2}\right]-E{\left[C_{\mathrm{inst}.}\right]}^{2}, \end{array}$$

where $E[C_{\mathrm{inst}.}^{2}]$ is given by Equation (A17) with $E\left[\log_{2}^{2}\left(\frac{\rho M\eta }{d_{k}^{2}}\right)\right]$ being defined by Equation (A18) with a=log(ρMη), $b=R-R_{min}$, c=log(R), $d=\log(R_{min)}$. Therefore, plugging Equations (A15) and (A17) into Equation (A16) we have Equation (A19). The proof is concluded by replacing Equations (A15) and (A19) into the definitions of κ and θ.

(A17) $$\begin{array}{cc} E[C_{\mathrm{inst}.}^{2}] & =K\left\{E\left[\log_{2}^{2}\left(\frac{\rho M\eta }{d_{k}^{2}}\right)\right]+\left(K-1\right)E{\left[\log_{2}\left(\frac{\rho M\eta }{d_{k}^{2}}\right)\right]}^{2}\right\}\\ \; & =K\left\{E\left[\log_{2}^{2}\left(\frac{\rho M\eta }{d_{k}^{2}}\right)\right]-E{\left[\log_{2}\left(\frac{\rho M\eta }{d_{k}^{2}}\right)\right]}^{2}\right\}\\ \; & =\frac{K\left(\frac{a^{2}b+4a\;\left(R-cR-R_{min}+dR_{min}\right)+8b+4cR\left(c-2\right)-4d\left(d-2\right)R_{min}}{b}-K^{2}{\left(a+\frac{2(R-cR-R_{min}+dR_{min})}{b}\right)}^{2}\right)}{\log^{2}\left(2\right)}. \end{array}$$

(A18) $$\begin{array}{cc} E\left[\log_{2}^{2}\left(\frac{\rho M\eta }{d_{k}^{2}}\right)\right] & =\int_{R_{min}}^{R}\log_{2}^{2}\left(\frac{\rho M\eta }{d_{k}^{2}}\right)\frac{1}{R-R_{min}}dr\\ \; & =\frac{a^{2}b+4a\;\left(R-cR-R_{min}+dR_{min}\right)+8b+4cR\left(c-2\right)-4d\left(d-2\right)R_{min}}{b\log^{2}\left(2\right)}. \end{array}$$

(A19) $$\sigma_{C_{\mathrm{inst}.}}^{2}=\frac{K\left(\frac{b\log^{2}\left(c\right)+4a\;\left(R-cR-R_{min}+dR_{min}\right)+8b+4c\left(c-2\right)R-4d\left(d-2\right)R_{min}}{b}-K\left(K+1\right){\left(a+\frac{2(R-cR-R_{min}+dR_{min})}{b}\right)}^{2}\right)}{\log^{2}\left(2\right)}$$
